# Design and Synthesis of a Cell-Permeable, Drug-Like Small Molecule Inhibitor Targeting the Polo-Box Domain of Polo-Like Kinase 1

**DOI:** 10.1371/journal.pone.0107432

**Published:** 2014-09-11

**Authors:** Ganipisetti Srinivasrao, Jung-Eun Park, Sungmin Kim, Mija Ahn, Chaejoon Cheong, Ky-Youb Nam, Pethaiah Gunasekaran, Eunha Hwang, Nam-Hyung Kim, Song Yub Shin, Kyung S. Lee, Eunkyung Ryu, Jeong Kyu Bang

**Affiliations:** 1 Division of Magnetic Resonance, Korea Basic Science Institute, Ochang, Chung-Buk, Republic of Korea; 2 Laboratory of Metabolism, Center for Cancer Research, National Cancer Institute, National Institutes of Health, Bethesda, Maryland, United States of America; 3 Institute for Innovative Cancer Research and Department of Convergence Medicine, Asan Medical Center, Seoul, Republic of Korea; 4 Molecular Embryology Laboratory, Department of Animal Sciences, Chungbuk National University, Cheongju, Chung-Buk, Republic of Korea; 5 Department of Bio-Materials, Graduate School and Department of Cellular & Molecular Medicine, School of Medicine, Chosun University, Gwangju, Republic of Korea; University of Bologna & Italian Institute of Technology, Italy

## Abstract

**Background:**

Polo-like kinase-1 (Plk1) plays a crucial role in cell proliferation and the inhibition of Plk1 has been considered as a potential target for specific inhibitory drugs in anti-cancer therapy. Several research groups have identified peptide-based inhibitors that target the polo-box domain (PBD) of Plk1 and bind to the protein with high affinity in in vitro assays. However, inadequate proteolytic resistance and cell permeability of the peptides hinder the development of these peptide-based inhibitors into novel therapeutic compounds.

**Methodology/Principal Findings:**

In order to overcome the shortcomings of peptide-based inhibitors, we designed and synthesized small molecule inhibitors. Among these molecules, bg-34 exhibited a high binding affinity for Plk1-PBD and it could cross the cell membrane in its unmodified form. Furthermore, bg-34-dependent inhibition of Plk1-PBD was sufficient for inducing apoptosis in HeLa cells. Moreover, modeling studies performed on Plk1-PBD in complex with bg-34 revealed that bg-34 can interact effectively with Plk1-PBD.

**Conclusion/Significance:**

We demonstrated that the molecule bg-34 is a potential drug candidate that exhibits anti-Plk1-PBD activity and possesses the favorable characteristics of high cell permeability and stability. We also determined that bg-34 induced apoptotic cell death by inhibiting Plk1-PBD in HeLa cells at the same concentration as PEGylated 4j peptide, which can inhibit Plk1-PBD activity 1000 times more effectively than bg-34 can in in vitro assays. This study may help to design and develop drug-like small molecule as Plk1-PBD inhibitor for better therapeutic activity.

## Introduction

**Figure 1:**
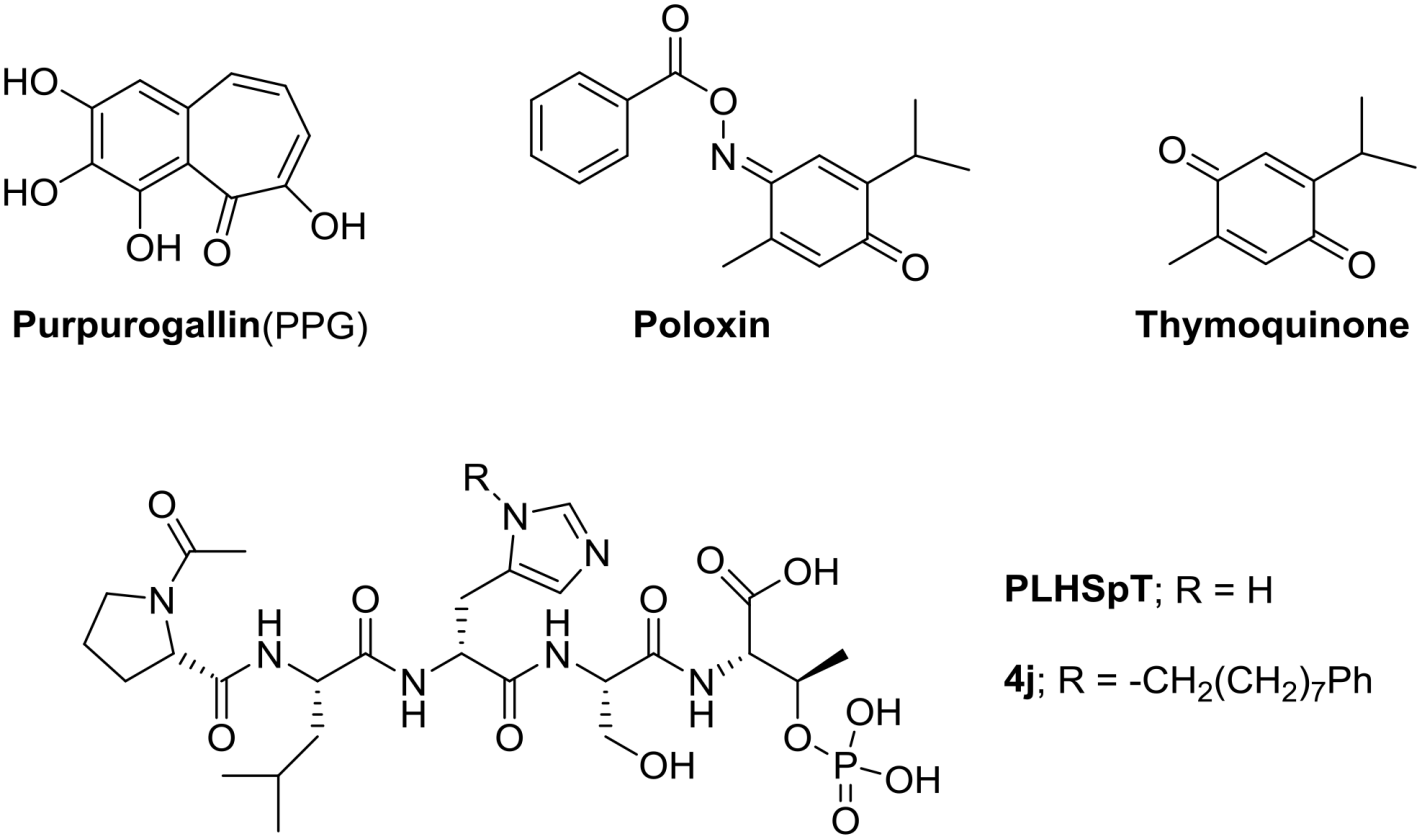
Structures of PPG, poloxin, thymoquinone, PLHSpT, and 4j.

Polo-like kinases (Plks) 1–4 play critical roles in numerous cell cycle-related activities including the initiation of mitosis, chromosome segregation, centrosome maturation, bipolar-spindle formation, regulation of the anaphase-promoting complex, and execution of cytokinesis [1]–[6] whereas the Plk5 does not appear to function in cell-cycle progression. Among five human Plks, Plk1 has been studied most extensively because its activity can override spindle checkpoints and induce genetic instability and thereby promote tumorigenesis [7]–[11]. Because the overexpression of Plk1 is strongly correlated with the aggressiveness and prognosis of several cancers [9], Plk1 has been examined as a potential target for specific inhibitory drugs in anti-cancer therapy. Plk1, which is a key regulator of mitotic progression and cell division in eukaryotes, possesses an *N*-terminal catalytic domain and a *C*-terminal polo-box domain (PBD) composed of two highly homologous polo boxes (PB1 and PB2). A large body of evidence suggests that the PBD recognizes phosphoserine (pS)/phosphothreonine (pT)-containing motifs with *N*-proximal serine residues (S-(pT/pS)), and directs the *N-*terminal catalytic domain for specific subcellular locations [12]–[14], which plays crucial in cell regulating mitotic progression and proliferation [15]–[17]. Therefore, targeting the PBD rather than kinase domain could be an alternative strategy to overcome the cross-reactivity commonly associated with kinase-domain inhibitors [18], [19]. Because of its essential role in cell proliferation, there has been a high level of interest to identify the interaction between Plk1 and its centromere/kinetochore-associated binding target, that led to the discovery of polo-box-interacting protein 1 (PBIP1) and a minimal peptide, PLHSpT (Figure 1) (residues 74–78 of PBIP1) [20] and its derivatives that exhibit high anti-Plk1-PBD activity [21]–[30]. However, peptide-based drugs are widely recognized to present severe shortcoming in terms of proteolytic stability and cell permeability, and this hampers the development of the peptide drugs into novel therapeutic agents. To enhance their cell permeability, these peptide inhibitors must be modified by, for example, conjugating them with a cell-penetrating peptide or PEGylating them, but this requires complicated synthetic steps and also occasionally results in a reduction of binding affinity.

To overcome the aforementioned drawbacks, cell-permeable, drug-like small molecules that target Plk1-PBD must be developed. In addition to various PBD-binding phosphopeptides, to date, three small molecules have been identified as Plk1-PBD inhibitors: purpurogallin (PPG) [17], poloxin [15], and thymoquinone [15] (Figure 1). However, we recently demonstrated that only PPG binds to Plk1-PBD and that its binding affinity was 6-times lower than that of PLHSpT [31].

To continue our efforts aimed at discovering and developing Plk1-PBD inhibitors [29], [30], in this study, we used a structure-based approach to design a novel, potent, cell-permeable small molecule named bg-34, which exhibits a high binding affinity for Plk1-PBD; this binding affinity was 5-fold higher than that of PLHSpT and 30-fold higher than that of PPG. Furthermore, to identify bg-34 as a potential drug that targets Plk1-PBD, we examined how bg-34 inhibits Plk1 function and how the drug is taken up by HeLa cells. Moreover, to investigate the nature of bg-34 binding to Plk1-PBD, we conducted molecular-modeling studies on Plk1-PBD in complex with bg-34. To the best of our knowledge, this is the first report to describe a small molecule as a promising drug candidate designed for targeting Plk1-PBD.

## Results and Discussion

### Structure-guided design of small molecules

To design a drug-like small molecule that inhibits Plk1, we first closely examined the nature of Plk1-PBD binding to and complex formation with phosphopeptide ligands. Until now, seven X-ray crystal structures of human Plk1-PBD have been determined [12], [13], [15], [32], and these crystal structures have shown that the peptides bind in a similar manner within a positively charged grove located between PB1 and PB2. Specifically, analysis of the crystal structure of Plk1-PBD in complex with a ligand revealed that three binding pockets are critical to bind Plk1-PBD with high affinity: (1) SpT-dependent electrostatic binding pocket in which the SpT motif of the phosphopeptide acting as a high-affinity anchor. (2) pyrrolidine binding pocket surrounded by Trp414, Phe535, and Arg516 residues in which *N*-terminal Pro residue provides additional binding affinity to the Plk1 PBD through participating in a hydrogen bonding interaction between carbonyl oxygen and the guanidium moiety of Arg516 of the PBD. (3) Tyr-rich channel that is surrounded by Tyr417, Tyr481 and Tyr485 in which long chain *n*-alkyl phenyl group anchored on His residue of phosphopeptide provides strong binding affinity through hydrophobic interactions with the above residues (Figure S1 in File S1) [16], [30].

These observations suggest that appropriate design of non-peptide, small-molecule scaffold would provide drug-like small molecule targeting Plk1-PBD with high binding affinity. To mimic all the above interacting residues of compound 4j, we presented a benzimidazole skeleton as our scaffold which provides rigidity around the central unit. This skeleton allows silent structural features of high affinity SpT, *n*-alkylphenyl His motif and specificity inducing *N*-terminal Pro residue as in compound 4j. Moreover, its derivatives play predominant role in drug discovery and can be regarded as highly favored structures in medicinal chemistry [33].

### First-phase synthesis

**Figure 2:**
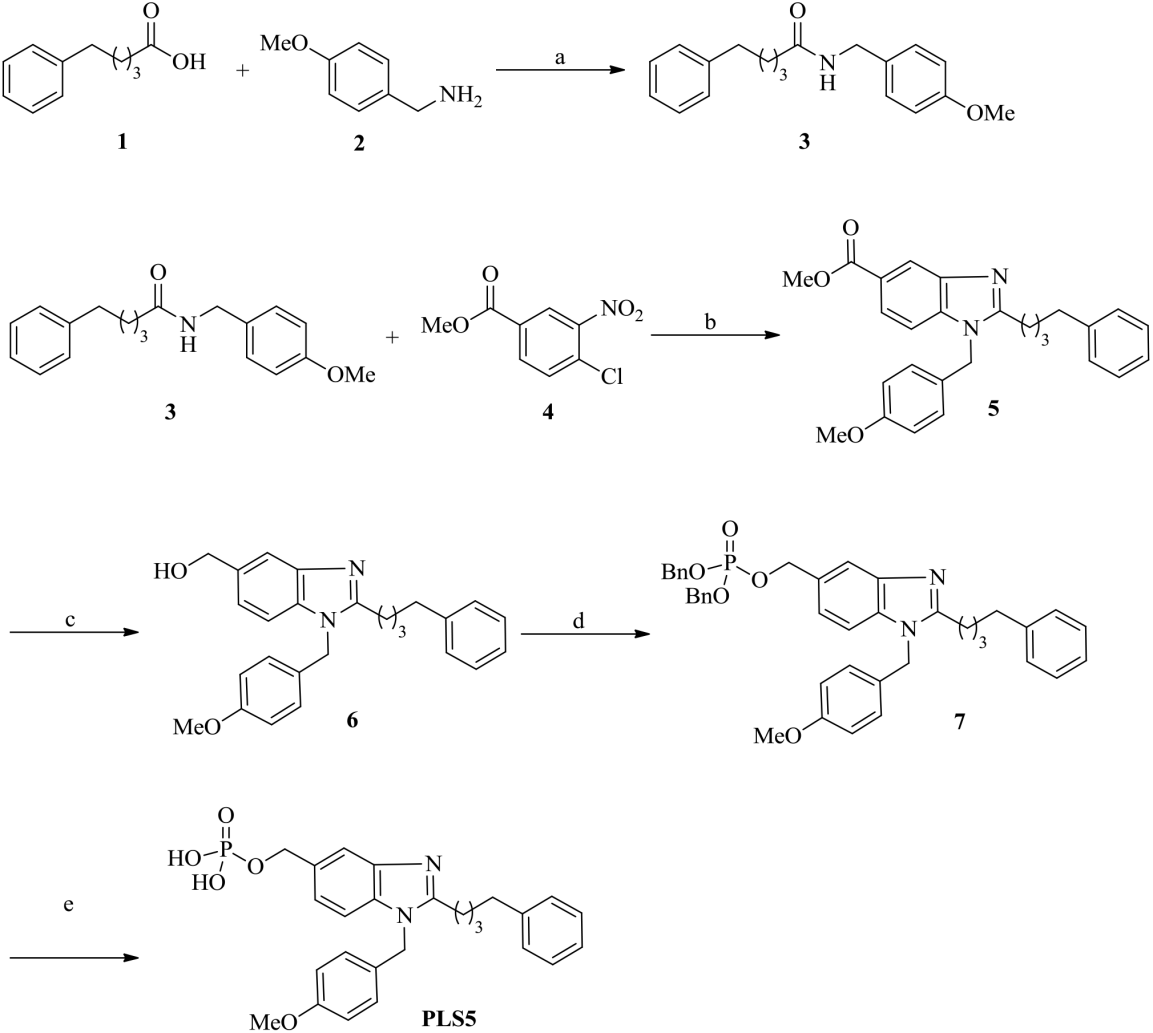
Reagents and conditions: (a) EDCI, HOBt, DIEA, CH_2_Cl_2_, room temp, 4 h, 90%; (b) (i) Pd(TFA)_2_, BINAP, Cs_2_CO_3_, toluene, 80°C, 8 h, and (ii) AcOH, Fe powder, reflux, 2 h (60% for 2 steps); (c) LiAlH_4_, THF, 0°C, 1 h, 90%; (d) dibenzyl-*N*,*N*-diisopropylphosphoramidite, 1*H*-tetrazole, DMF, 0°C to room temp, 2 h, and then *^t^*BuOOH, 0°C, 1 h, 85%; (e) TFA, triisopropylsilane, H_2_O, 2 h, 90%.

We started the inhibitor synthesis by using the commercially available compounds 5-phenyl valeric acid **1** and (4-methoxyphenyl) methanamine **2** (Figure 2). The reaction of these two compounds in the presence of 1-ethyl-3-(3-dimethylaminopropyl) carbodiimide (EDCI), 1*-*hydroxybenzotriazole (HOBt) and *N,N*-diisopropylethylamine (DIEA) yielded the amide compound **3**. Treating the amide **3** with methyl 4-chloro-3-nitrobenzoate **4** in the presence of Pd(TFA)_2_, BINAP, and Cs_2_CO_3_ and then refluxing with AcOH/Fe generated the benzimidazole derivative **5**, which is a key intermediate in our synthetic process [34]. The ester functional group in **5** was reduced using LiAlH_4_ to generate the alcohol **6**. The alcohol functional group in **6** was phosphorylated by using dibenzyl-*N*,*N*-diisopropylphosphoramidite in the presence of ^1^ *H*-tetrazole, and then it was oxidized using *tert-*butyl hydroperoxide (*^t^*BuOOH) to create the corresponding phosphoric ester **7**. Finally, deprotection of benzyl groups in **7** by using TFA generated the molecule PLS5 (Figure 2) [35].

**Figure 3:**
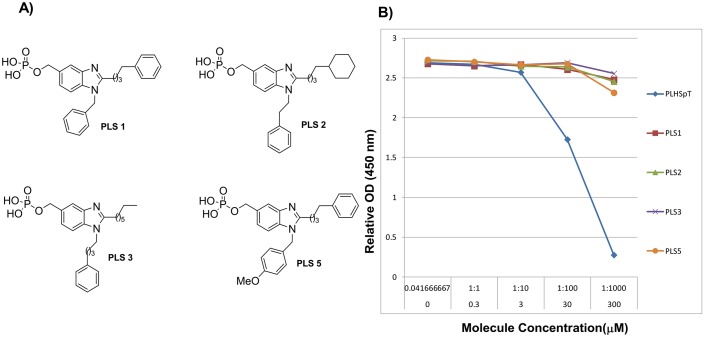
Structures of PLS1–PLS3 and PLS5 (A). ELISA-based measurement of Plk1-PBD binding (B); the IC_50_ graph is shown (O.D., optical density).

Using the aforementioned strategy, we synthesized PLS1–PLS3, in which distinct alkyl groups were tethered on the imidazole ring to target the Tyr-rich channel and the pyrrolidine-binding pocket of Plk1-PBD (Figure 3A).

Next, we focused on the Tyr-rich channel of Plk1-PBD, which is surrounded by Tyr417, Tyr481, and Tyr485; to target this channel, we incorporated a butylphenyl chain on the imidazole ring of PLS1, because our recent results revealed that the phenyl ring featuring four-carbon tether was efficient for reaching the Tyr-rich channel of Plk1-PBD [30]. Furthermore, to examine the effect of aromaticity by π-π stacking interactions with the Tyr-rich channel, we incorporated nonaromatic groups, which included cyclohexane in PLS2 and an alkyl chain in PLS3. Finally, we examined the pyrrolidine-binding pocket of Plk1-PBD, which is broad and shallow and is surrounded by Trp414 and Phe535 [16]. To enable binding with the pyrrolidine-binding pocket by means of π-π stacking interactions, we incorporated an aromatic (phenyl) group at *N*-position of bezimidazole featuring distinct carbon tethers in PLS1–PLS3 and PLS5.

### Preliminary evaluation

The competitive inhibitory activity of the synthesized compounds PLS1–PLS3 and PLS5 were evaluated using an ELISA-based Plk1-PBD-inhibition assaydescribed previously [16]. The assay results revealed that PLS1–PLS3 did not show any binding affinity even at the high concentration. In contrast, PLS5, which features the same configuration as PLS1 except for the presence of a methoxy group on the phenyl ring, showed weak binding to Plk1-PBD at high concentration (Figure 3B). Based on the preliminary assay results, PLS5 became a core skeleton to further improve in binding affinity against Plk1-PBD.

### Second-phase synthesis

Since PLS5 binds to Plk1-PBD weakly, we investigated to enhance binding affinity of this molecule. First, we closely examined the phospho-binding pocket of Plk1-PBD, which is critical for obtaining high-binding affinity. The phosphate group in PLS5 scaffold resembles a pSer and moreover, Elia, A. E *et al*. described that replacing pThr with pSer in the peptide lowered the peptide’s binding affinity for Plk1-PBD [12]. This finding suggested that adding a pThr group instead of a phosphate group (a pSer mimic) would increase the binding affinity of PLS5 for Plk1-PBD.

**Figure 4:**
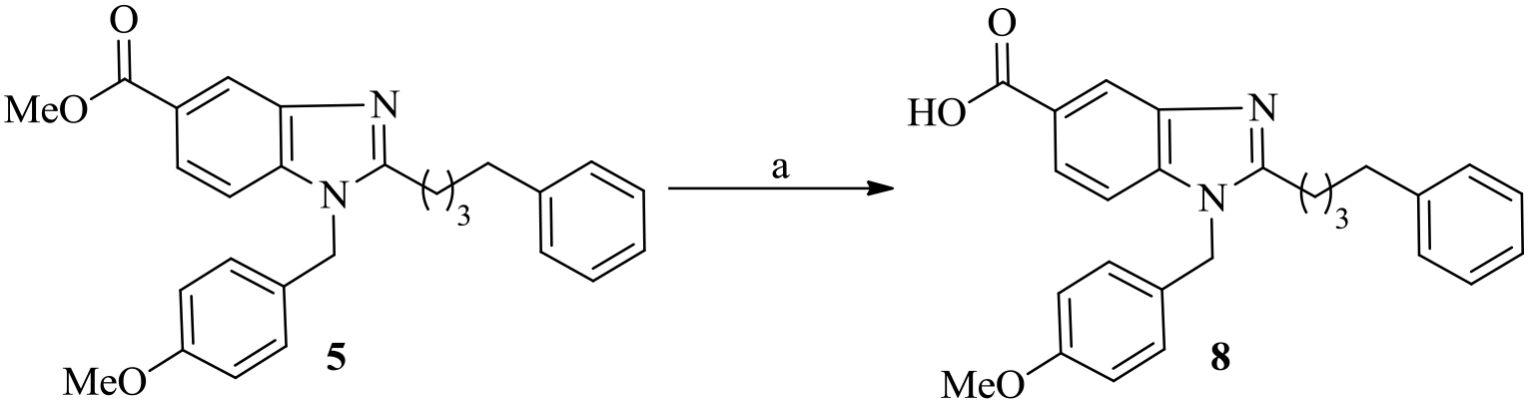
Reagents and conditions: (a) 1,4-dioxane, 4N HCl, reflux, 2 h, 80%.

**Figure 5:**
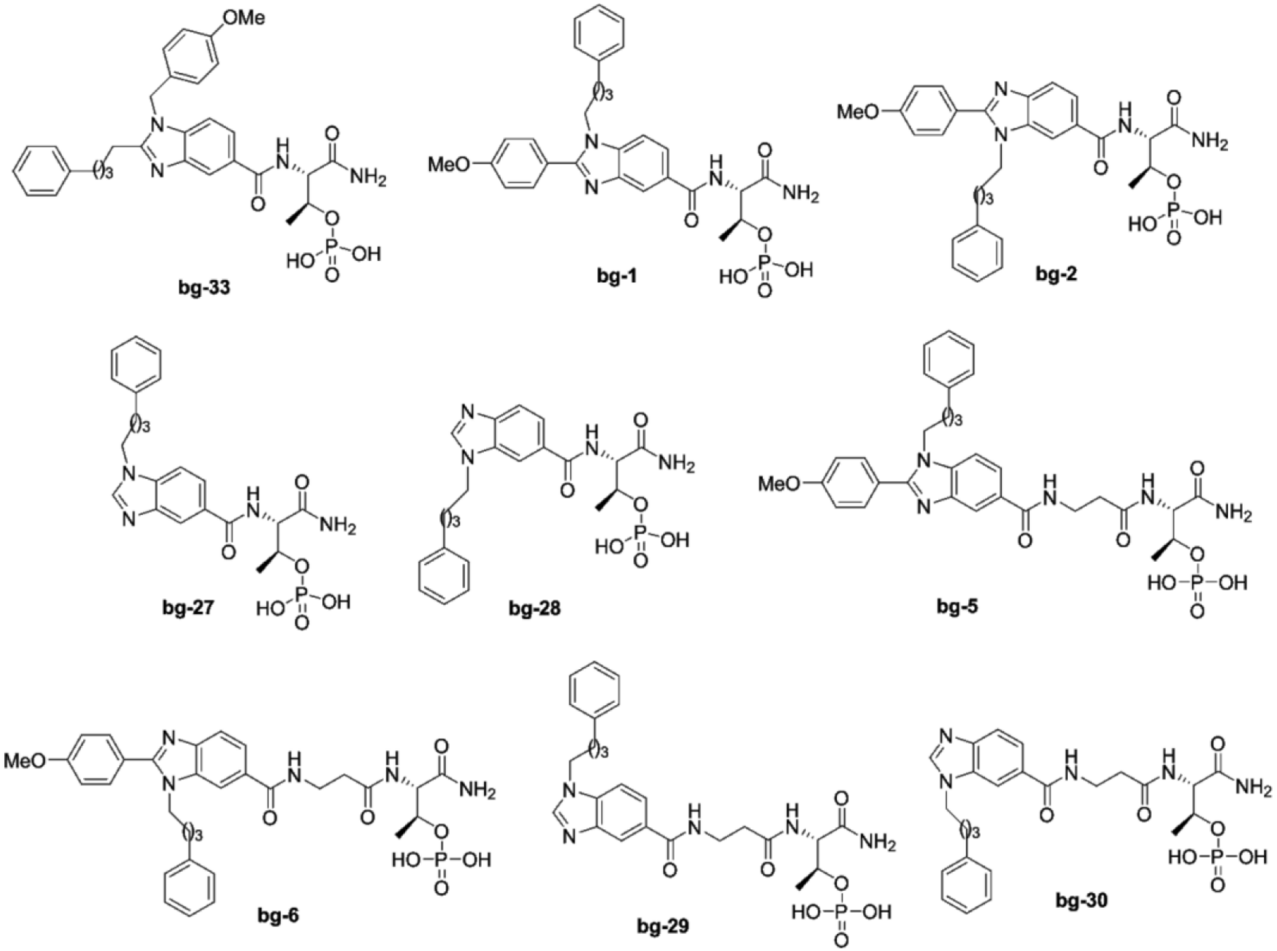
Structures of bg-33, bg-1, bg-2, bg-27, bg-28, bg-5, bg-6, bg-29, and bg-30.

**Figure 6:**
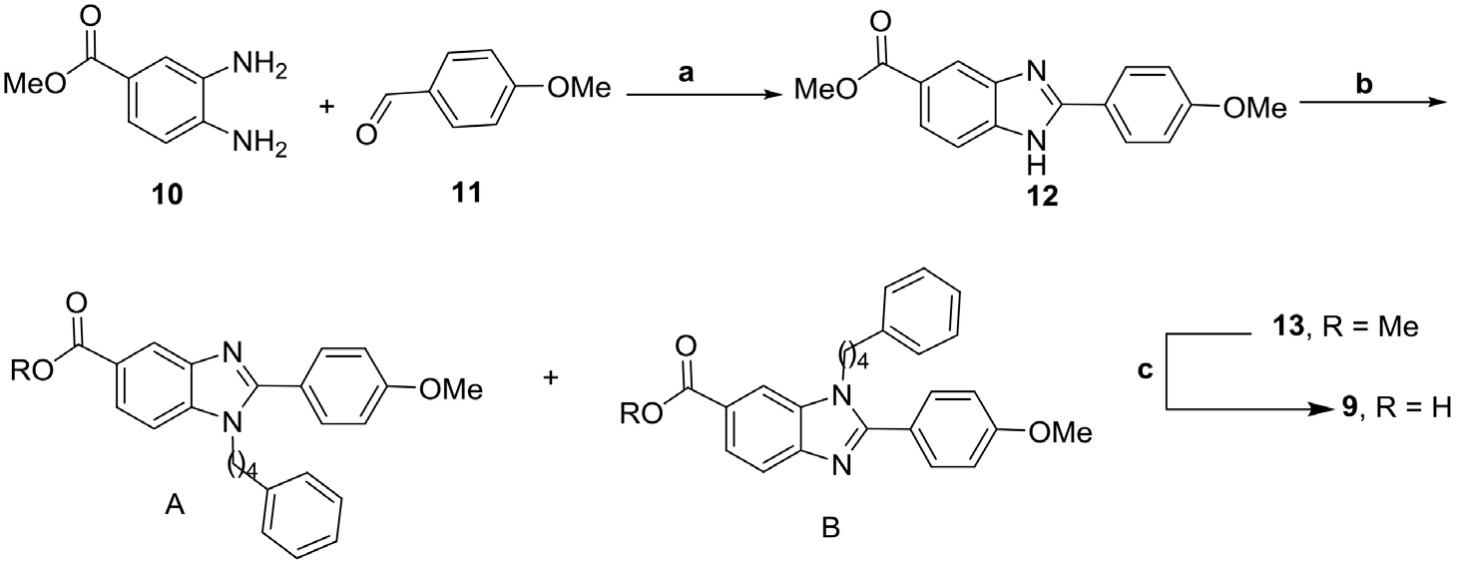
Synthesis of 9: Reagents and conditions: (a) *p*-TsOH (cat), DMF, 60°C, 2 h, 70%; (b) 4-phenyl butyl bromide, K_2_CO_3_, DMF, 80°C, 2 h, 90%; (c) 1,4-dioxane, 4N HCl, reflux, 2 h, 85%.

As stated above, to increase the binding affinity of Plk1-PBD by interacting with the electrostatic binding pocket surrounded by positively charged His538 and Lys540 residues, we synthesized bg-33 by starting from the ester **5** (Figure 4). The ester functional group in **5** was hydrolyzed using 1,4-dioxane in the presence of 4N HCl under reflux conditions; this yielded the acid derivative **8**, which was used for solid-phase synthesis of the molecule bg-33 by using pThr in the presence of the coupling agents 1-*O*-Benzotriazole-*N*,*N*,*N*′,*N′*-tetramethyluronium hexafluoro-phosphate (HBTU), HOBt, and DIEA (Figure 5). Next, to examine the effect of alkyl-chain position on the molecule’s binding affinity for Plk1-PBD, we synthesized bg-1 and bg-2 which are positional isomers of bg-33 (Figure 5). In this synthetic process, we first synthesized the acid derivative **9** by starting from methyl 3,4-diaminobenzoate **10**. The diamine **10** was treated with anisaldehyde **11** in the presence of catalytic anhydrous *p*-TSA in DMF at 80°C, which generated the key intermediate **12** (Figure 6) [36]. The *n*-alkylation of **12** by using 4-phenyl butyl bromide in the presence of K_2_CO_3_ in DMF at 60°C produced the compound **13** as an isomeric mixture of **13a** and **13b**. The *n*-alkylated mixture **13** was hydrolyzed by using 1,4-dioxane in the presence of 4 N HCl under reflux conditions, which yielded the acid **9** as a non-separable isomeric mixture of **9a** and **9b**; from this mixture, we synthesized bg-1 and bg-2 by using Thr(PO(OBzl)OH)-OH in the presence of HBTU, HOBt, and DIEA, which served as coupling agents.

**Figure 7:**
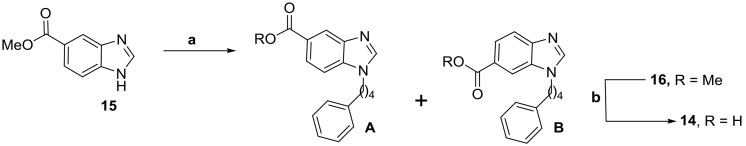
Synthesis of 14: Reagents and conditions: (a) 4-phenyl butyl bromide, K_2_CO_3_, DMF, 80°C, 2 h, 90%; (b) 1,4-dioxane, 4N HCl, reflux, 2 h, 85%.

We speculate that the butylphenyl group and the methoxy phenyl group on the benzimidazole ring may block each other which hinders alkyl residues to reach the proper binding channels in Plk1-PBD effectively. In an attempt to eliminate this problem, we synthesized the mono-alkylated analogues such as bg-27 and bg-28 (Figure 5). To synthesize bg-27 and bg-28, we initially synthesized the acid derivative **14** by starting from methyl 1*H*-benzo[*d*]imidazole-5-carboxylate **15**. The compound **15** was *n*-alkylated by using 4-phenyl butyl bromide in the presence of K_2_CO_3_ in DMF at 60°C, and this generated the compound **16** as an isomeric mixture of **16a** and **16b**. The ester functional group in **16** was hydrolyzed by using 1,4-dioxane in the presence of 4N HCl under reflux conditions, which yielded the acid **14** as an isomeric mixture of **14a** and **14b** (Figure 7); from this mixture, we synthesized bg-27 and bg-28 using Thr(PO(OBzl)OH)-OH in the presence of the coupling agents HBTU, HOBt, and DIEA.

To investigate the effect of extending the chain length between the pThr group and the benzimidazole, we synthesized bg-5, bg-6, bg-29 and bg-30 by using *β*-alanine. So there may be a feasibility of butylphenyl chain on the imidazole ring to reach the proper binding channel in Plk1-PBD (Figure 5).

### Second-phase results of Plk1 PBD binding affinity

ELISA-based inhibition-assay results indicated that bg-33 completely lost its binding ability against Plk1-PBD because alkyl groups on imidazole ring in bg-33 may not direct in proper binding channels. In addition, the molecules bg-1 and bg-2 which are positional isomers of bg-33 also did not exhibit any binding affinity for Plk1-PBD. These results support our assumption that both alkyl groups on benzimidazole ring in bg-33 as well as bg-1 and bg-2 might be blocking each other. To investigate this problem, we examined bg-27 and bg-28 and the assay result of these two molecules did not exhibit any binding affinity for Plk1-PBD. From these results, we concluded that the alkylphenyl groups collision was not the prime factor for the failure of bg-1 and bg-2 to exhibit good binding. Next, we tested bg-5, bg-6, bg-29 and bg-30 which increase the distance between the pThr group and the benzimidazole ring. Assay results showed that bg-5 and bg-6 bind to Plk1 PBD weakly. However, bg-29 and bg-30 did not exhibit any binding activity at the tested concentration.

**Figure 8:**
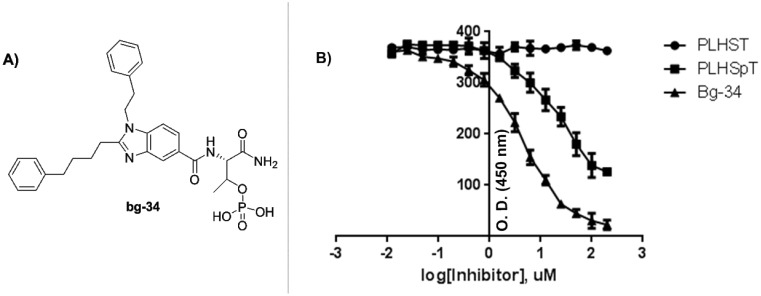
Structure of bg-34 (A) and ELISA-based measurement of Plk1-PBD binding (B); the IC_50_ graph is shown (O.D., optical density).

**Figure 9:**
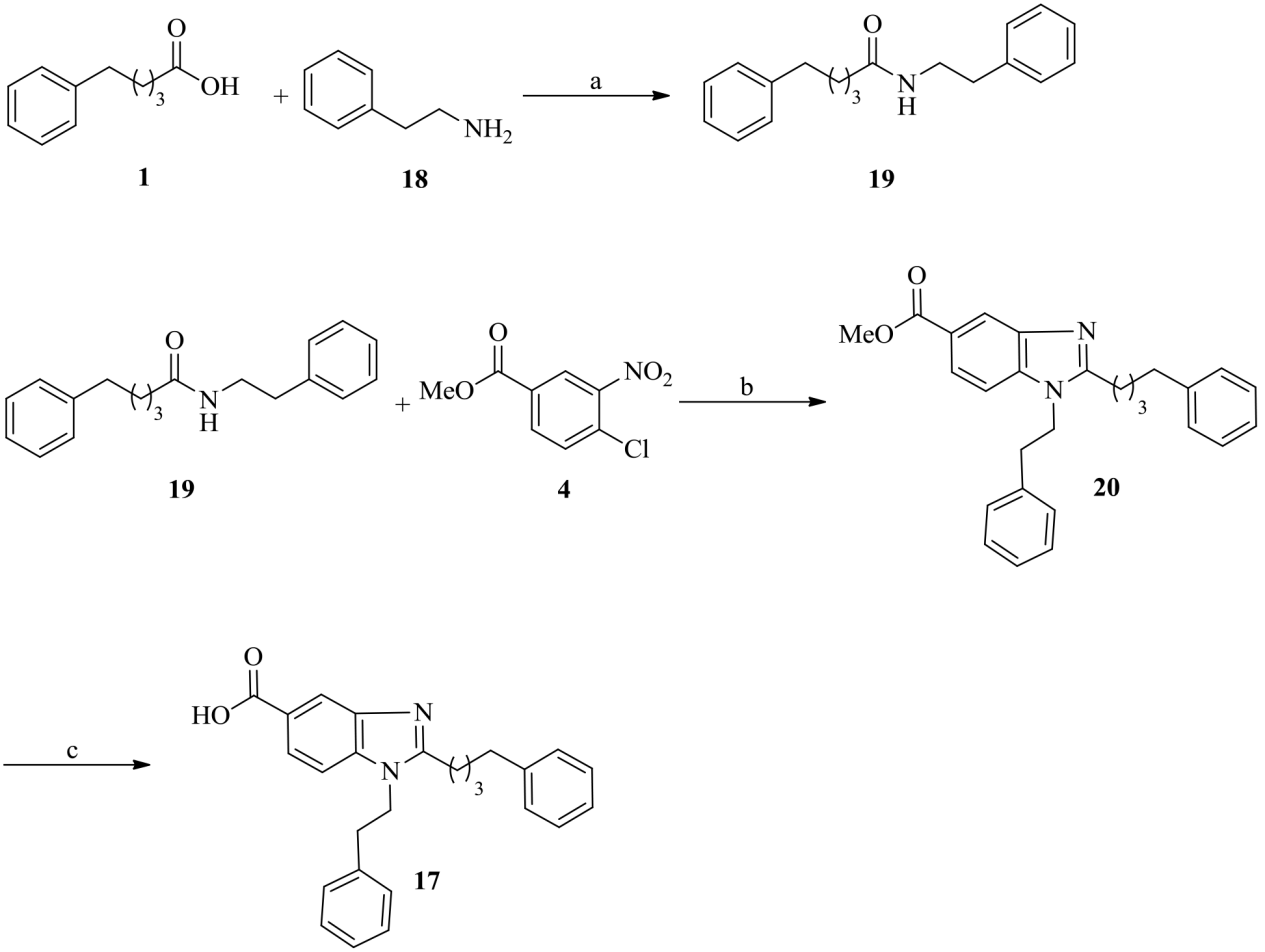
Reagents and conditions: (a) EDCI, HOBt, DIEA, CH_2_Cl_2_, room temp, 4 h, 90%; (b) (i) Pd(TFA)_2_, BINAP, Cs_2_CO_3_, toluene, 80°C, 8 h, and (ii) AcOH, Fe powder, reflux, 2 h (60% for 2 steps); (c) 1,4-dioxane, 4N HCl, reflux, 2 h, 80%.

It was well documented that Tyr-rich channel of Plk1-PBD was extremely narrow and deep, whereas the pyrrolidine-binding pocket was broad and shallow [16]. These findings indicated that small molecules must possess long tethers to interact with the Tyr-rich channel and they must be flexible to interact with the pyrrolidine-binding pocket. We surmised that in bg-33, the four-carbon tether on the phenyl group would be adequate for interacting with the Tyr-rich channel, but that the methoxy phenyl group would be flexible if we increased the distance between the phenyl and imidazole rings. Based on considering all these factors, we synthesized the molecule bg-34 (Figure 8A) by using the compound **17**, the synthesis of which was started from 5-phenyl valeric acid (**1**) and 2-phenylethanamine **18** (Figure 9). The reaction of **1** and **18** in the presence of EDCI, HOBt, and DIEA generated the amide **19**, which was treated with methyl 4-chloro-3-nitrobenzoate in the presence of Pd(TFA)_2_, BINAP, and Cs_2_CO_3_ and then refluxed with AcOH/Fe to generate the benzimidazole derivative **20**. Hydrolysis of the ester **20** with 1,4-dioxane in the presence of 4N HCl under reflux conditions yielded the acid **17**, and bg-34 was synthesized from the acid derivative **17** by using Thr(PO(OBzl)OH)-OH in the presence of HBTU, HOBt, and DIEA.

In striking contrast to the results obtained for other aforementioned molecules, our Plk1-inhibition assay results revealed that bg-34 exhibited extremely high binding affinity for Plk1-PBD and we determined its IC_50_ to be 4.53 µM, which is approximately 5 times more than that of PLHSpT (IC_50_ = 23.42 µM) (Figure 8B). These results support our hypothesis that the three functional groups and the scaffold of bg-34 together determine high-affinity binding by interacting with the phospho-binding pocket, the Tyr-rich channel, and the pyrrolidine-binding pocket of Plk1-PBD.

Relative activities of the compounds PLHSpT, PPG, PLSn (n = 1, 2, 3 & 5), Bgn (n = 1, 2, 5, 6, 27–30, 33 & 34) in the inhibition of the Plk1 PBD are summarized in Table S1 in File S1.

### Molecular modeling

**Figure 10:**
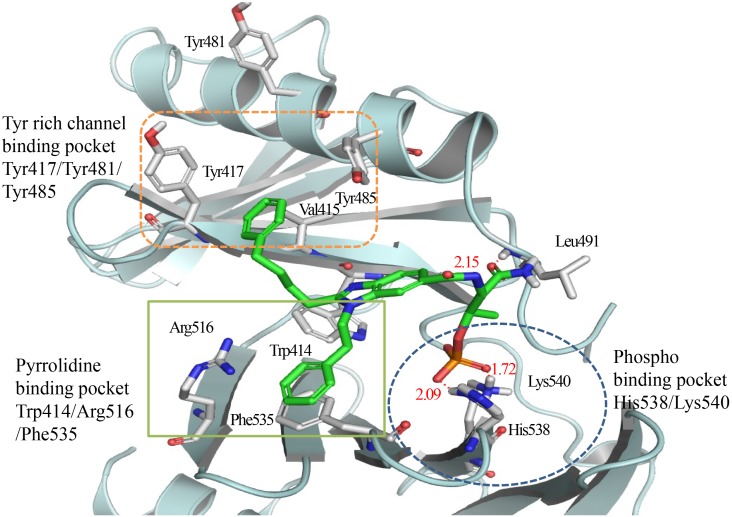
Modeled structure of Plk1-PBD in complex with bg-34; the model shows the presence of the phosphate-binding pocket, the pyrrolidine-binding pocket, and the Tyr-rich hydrophobic channel. The model was generated using Pymol (http://www.pymol.org).

To enhance our understanding of the nature of bg-34 binding to Plk1-PBD, we conducted molecular-modeling studies on Plk1-PBD in complex with bg-34 (Figure 10). This modeling work also supported our hypothesis that both the benzimidazole scaffold and the three functional groups were required for strong interaction with Plk1-PBD. The model suggests that the negatively charged phosphate group of bg-34 interacts with the positively charged residues Lys540 and His538, which are located at the phospho-binding pocket. The long-chain phenyl group (butylphenyl) introduced in bg-34 entered the narrow passage of the Tyr-rich hydrophobic channel that is surrounded by Tyr485, Val415, and Tyr417 and engaged in hydrophobic interactions, and the short-chain phenyl group (ethyl phenyl) substituted in bg-34 additionally binds to the pyrrolidine-binding pocket by means of *π*-*π* stacking interactions with Trp414 and Phe535. From the modeling study of bg-34, we tried to explain inefficiency of bg-1, bg-2, bg-27 and bg-28 to show binding affinity with the Plk1 PBD. In case of bg-1 and bg-2, methoxy phenyl group could not reach the pyrrolidine binding pocket because there is no two carbon linker between methoxy phenyl and benzimidazole groups. This hypothesis was supported by increasing activity of bg-34 which has two carbon linker between phenyl group and benzimidazole group. The lost binding affinity in bg-27 and bg-28 implied that two functional groups are not enough to interact with Plk1 PBD using our benzimidazole scaffold. The above observations suggest that three functional groups are essential for achieving the effective interaction with Plk1 PBD in terms of Tyr-rich channel, pyrrolidine binding pocket and phospho binding pocket. To confirm binding mode of bg-34, we are also ongoing X-ray complex structure PBD with bg-34. We expect that X-ray complex structure will also support our hypothesis that bg-34 has mono-specificity against Plk1-PBD from closely related Plk2, and Plk3.

### Cellular uptake

Although phosphopeptides bind to Plk1-PBD with high affinity in vitro, the peptides are inefficiently taken up by cancer cell lines. To increase the cellular uptake of inhibitors, the phosphopeptides must be conjugated with a cell-penetrating peptide or they must be PEGylated; however, these methods are time-consuming and require advanced skills, and this raises the cost of developing anti-Plk1-PBD therapeutic agents. Furthermore, these strategies occasionally cause the inhibitors to lose their Plk1-PBD-binding activity partially or completely. In light of these findings, we tested whether bg-34 is taken up by HeLa cells by performing fluorescence imaging; to examine the cellular uptake of bg-34, we conjugated it with fluorescein 5(6)-isothiocyanate (FITC) (as shown in Figure S2 in File S1) and then incubated 200 µM FITC-bg-34 with HeLa cells for 3 h.

**Figure 11:**
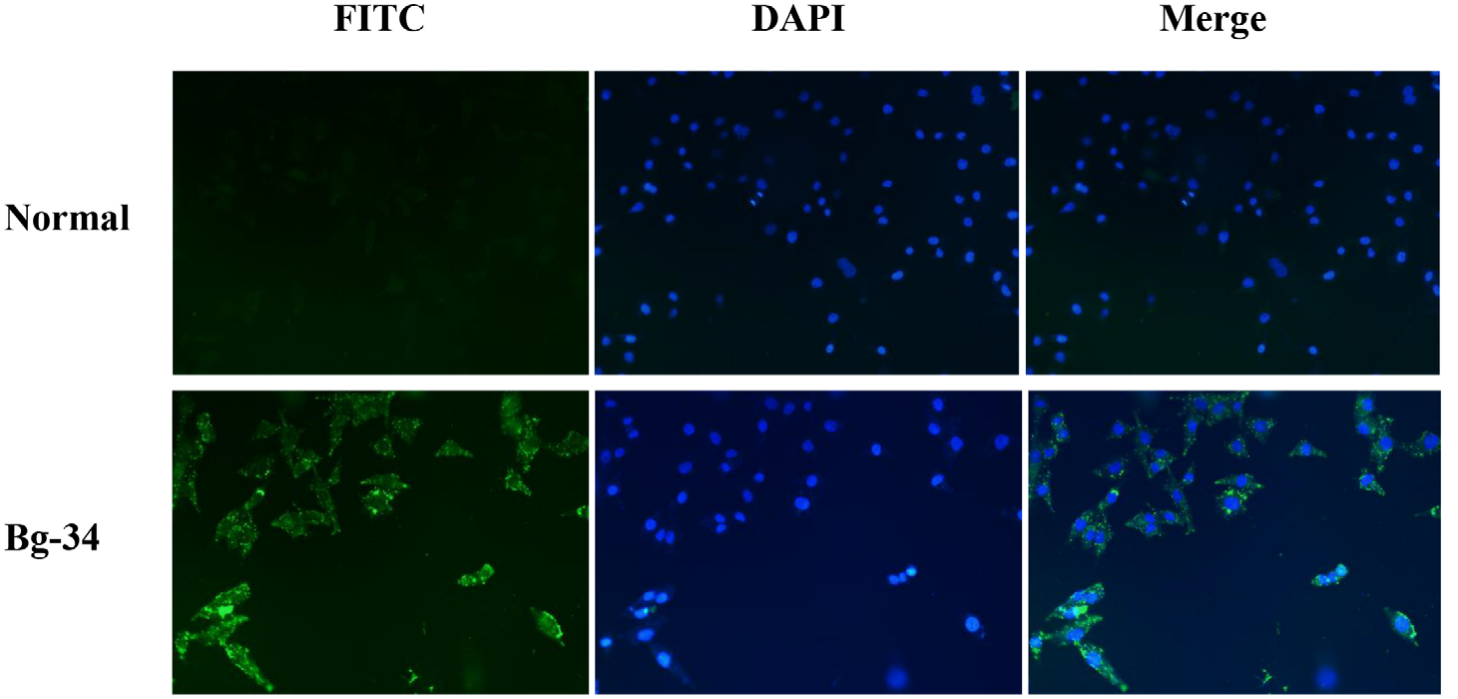
Fluorescence-microscopy images showing the uptake of FITC-conjugated bg-34 by HeLa cancer cells. Nuclei were stained with DAPI (blue) and bg-34 was detected by means of FITC fluorescence (green) and the images were overlaid; 200× magnification.

The results of the cellular-uptake assays showed that FITC alone (control) was not taken up by HeLa cells; by contrast, the cellular uptake of FITC-bg-34 was clearly observed (Figure 11). The fluorescence distribution in HeLa cells indicated that bg-34 was localized within discrete vesicular compartments of the cells, which suggested that endocytosis was the predominant mechanism by which bg-34 entered cells. This result implied that bg-34 overcome one of the main drawback of peptide-based drugs such as cellular impermeability.

### Inhibition of Plk1-PBD function by bg-34

**Figure 12:**
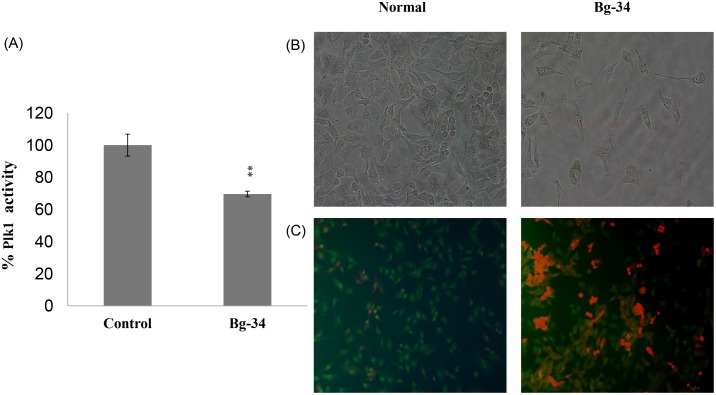
Inhibition of Plk1 kinase by bg-34 (A). HeLa cells were incubated with 200 µM bg-34 or with PBS and the effect of bg-34 treatment on the cancer cells was examined. Morphology of HeLa cells after incubation for 24 h with 200 µM bg-34 (B). Apoptosis in HeLa cells; apoptosis was detected by means acridine-orange staining after treatment with bg-34 for 24 h (200× magnification) (C).

To investigate whether bg-34 inhibits the kinase activity of Plk1, we tested the effect of the molecule directly on cultured HeLa cells. The results showed that after treatment with bg-34, Plk1 activity was lowered to approximately 65% of the activity in control, which implies that around 35% of the activity was inhibited (Figure 12A). Furthermore, to examine whether the uptake of bg-34 by the cells is sufficient for causing apoptosis through Plk1 inhibition, we incubated cultured HeLa cells with 200 µM bg-34 for 12 h. Unexpectedly, in the absence of any carriers, bg-34 potently induced apoptotic cell death and also caused a considerable degree of necrosis in HeLa cells, whereas the control PBS treatment did not exert any inhibitory effect (Figure 12B and 12C). The most potent peptide analog available for inhibiting Plk1 is **4j** (Figure 1) (IC_50_ = 17 nM), which must be PEGylated to make it cell permeable, but this modification drastically diminishes the ability of **4j** to inhibit Plk1 (≈200 µM) in cultured HeLa cells [16]; by comparison, in the cell-based assay, the inhibitory effect of bg-34 was substantially higher (IC_50_ = 4.5 µM). Although less potent than **4j** at inhibiting Plk1 in vitro, bg-34 exhibited superior pharmacokinetic properties, particularly enhanced membrane permeability and stability; thus, a greater fraction of the administered dose of bg-34 than of **4j** was available for producing biological effects. Therefore, although both bg-34 and PEGylated **4j** can inhibit Plk1, the synthesis of PEGylated **4j** requires greater time and expense than bg-34 synthesis and PEGylation also occasionally leads to a reduction of the inhibitor’s binding affinity for Plk1; all of these drawbacks were overcome with the design and use of bg-34.

## Conclusions

We have demonstrated that bg-34 is a promising drug-like small molecule that can bind to Plk1-PBD with high affinity and, moreover, can be efficiently taken up by cells even when it is in an unmodified state. We have also demonstrated that bg-34-dependent inhibition of Plk1 activity is sufficient for inducing apoptosis in HeLa cells. Furthermore, the results of molecular-modeling studies performed on Plk1-PBD in complex with bg-34 support our hypothesis that the scaffold used in the compound is suitable for enabling its effective binding to Plk1-PBD. This study could thus help guide the design and development of drug-like small molecules that function as Plk1-PBD-specific inhibitors and exhibit high therapeutic activity.

## Materials and Methods

All reactions requiring anhydrous conditions were conducted in flame-dried reaction vessels under a positive pressure of Argon. Reagents were obtained commercially (Sigma-Aldrich or TCI) and used without further purification. For the chromatography analysis, high-performance liquid chromatography (HPLC) grade solvents such as hexane, ethyl acetate, methylene chloride, and methanol were used. Thin layer chromatography (TLC) was performed on analytical Merck silica gel 60 F254 and flash chromatography was performed on Merck silica gel 60 (230–400 mesh). Proton, carbon-13 and phosphorus-31 NMR spectra were acquired on a Bruker Avance-300 MHz spectrometer operating at 300, 75 and 121 MHz, respectively. ^1^H NMR data are expressed as chemical shifts in ppm followed by multiplicity (s–singlet; d–doublet; t–triplet; q–quartet; m–multiplet), number of proton(s), and coupling constant(s) *J* (Hz). ^13^C NMR chemical shifts are expressed in ppm. The molecular masses of purified compounds were determined using matrix assisted laser-desorption ionization time-of-flight mass spectrometry (MALDI-TOF MS) (Shimadzu, Japan). Reverse-phase HPLC analysis (RP-HPLC) was carried out at 230 nm on an Agilent HPLC system equipped with a C_18_ analytical column (25010 mm, 5 micron). Two different linear gradients of 0.05% aq. TFA (eluent A) and 0.05% TFA in CH_3_CN (eluent B) were used with a flow rate of 1.5 mL/min at 25°C.

### Synthesis of *N*-(4-methoxybenzyl)-5-phenylpentanamide 3

5-phenylpentanoic acid **1** (1 g, 5.6 mmol) and methoxybenzylamine **2** (0.73 mL, 5.6 mmol) were stirred in the presence of coupling agents EDCI (1.12 g, 6.2 mmol) HOBt (0.84 g, 6.2 mmol) and DIEA (2.34 mL, 16.8 mmol) for 1 h in dry DCM, After completion of the reaction, mixture was quenched with H_2_O (5 mL) and extracted with DCM (250 mL). The combined organic layers were washed with saturated citric acid solution (20 mL), saturated NaHCO_3_ solution (20 mL) and brine. The organic phase was dried over anhydrous Na_2_SO_4_ and the solvent was evaporated. The resulted residue was purified by silica gel column chromatography using (ethyl acetate/hexane = 2∶3) yielded amide **3** (1.95 g, 90%) as liquid.

#### Spectral data

^1^H NMR (CDCl_3_, 300 MHz): *δ* 7.29–7.23 (m, 2H), 7.20–7.13 (m, 5H), 6.84 (d, 1H, *J* = 6.0 Hz), 5.73–5.67 (br s, 1H), 4.34 (d, 2H, *J* = 6.0 Hz), 3.78 (s, 3H), 2.61 (d, 2H, *J* = 5.7 Hz), 2.19 (d, 2H, *J* = 5.4 Hz), 1.73–1.60 (m, 4H).; ^13^C NMR (CDCl_3_, 75 MHz): 172.8, 158.8, 142.1, 130.6, 129.0, 128.3, 128.2, 125.7, 113.9, 55.2, 42.8, 36.3, 35.6, 31.0, 25.4.

### Synthesis of Methyl 1-(4-methoxybenzyl)-2-(4-phenylbutyl)-1H-benzo[*d*]imidazole-5-carboxylate 5

The compound **3** was reacted with methyl 4-chloro-3-nitrobenzoate (0.2 g, 0.93 mmol), (0.55 g, 1.12 mmol), in the presence of palladium trifluoroacetate (30 mg, 0.04 mmol), (*R*)-BINAP (56 mg, 0.04 mmol), and cesium carbonate (493 mg, 0.7 mmol) in dry toluene. The mixture was heated to 80°C under argon atmosphere and stirred for 12 h. After completion, reaction mixture was quenched with water (3 mL) and filtered through a pad of Celite. The filterate was washed with ethyl acetate (3×6 mL) and the collected organic layer was dried and evaporated. The resulted residue was dissolved in glacial acetic acid (20 mL) and was heated to reflux for 1 h in the presence of iron powder (0.63 g). The acetic acid was removed under vacuum, and the residue was suspended in saturated NaHCO_3_ and extracted with ethyl acetate (250 mL). The combined organic layer was washed with brine and dried over anhydrous Na_2_SO_4._ The resulted crude residue was purified using silica gel column chromatography (ethyl acetate/hexane = 2∶3) to yield the desired product **5** (0.23 g, 60% for two steps) as liquid.

#### Spectral data

^1^H NMR (CDCl_3_, 300 MHz): δ 8.46 (s, 1H); 7.93 (d, 1H, *J* = 6.0 Hz); 7.27–7.22 (m, 3H); 7.18–7.11 (m, 3H); 6.94 (d, 1H, *J* = 9.0 Hz), 6.81 (d, 1H, *J* = 6.0 Hz), 5.24 (s, 2H), 3.92 (s, 3H), 3.76 (s, 3H), 2.86 (t, 2H, *J* = 6.0 Hz), 2.62 (t, 2H, *J* = 6.0 Hz), 1.92–1.84 (m, 2H); 1.76–1.68 (m, 2H); ^13^C NMR (CDCl_3_, 75 MHz): 167.6, 159.3, 157.0, 142.2, 142.0, 138.6, 128.3, 128.2, 127.4, 127.3, 125.7, 124.2, 124.0, 121.5, 114.4, 109.2, 55.2, 52.0, 46.6, 35.5, 31.0, 27.5, 27.0.

### Synthesis of (1-(4-methoxybenzyl)-2-(4-phenylbutyl)-1H-benzo[*d*]imidazol-5-yl)methanol 6

LiAlH_4_ (10 mg, 0.24 mmol) in dry THF (10 mL) was added to the solution of compound **5** (0.2 g, 0.48 mmol) in THF (15 mL) at 0°C. The resulted reaction mixture was brought to room temperature and stirred for 1 h. After completion, reaction mixture was quenched with water and 15% NaOH solution at 0°C. The resultant white precipitate was filtered and washed with ethyl acetate (20 mL). The filtrate was concentrated by *vacuo* and purified by silica gel column chromatography (MeOH/DCM = 1∶49) to afford alcohol **6** (0.17 g, 90%) as a liquid.

#### Spectral data

^1^H NMR (CDCl_3_, 300 MHz): *δ* 7.69 (s, 1H), 7.23 (t, 3H, *J* = 6.0 Hz), 7.13 (dd, 4H, *J* = 6 and 15 Hz), 6.92 (d, 2H, *J* = 6 Hz), 6.79 (d, 2H, *J* = 6 Hz), 5.20 (s, 2H), 4.74 (s, 2H), 3.74 (s, 3H), 2.83 (t, 2H, *J* = 6.0 Hz), 2.59 (t, 2H, *J* = 6.0 Hz), 1.84 (q, 2H, *J* = 6.0), 1.69 (q, 2H, *J* = 6.0).; ^13^C NMR (CDCl_3_, 75 MHz): 159.2, 155.6, 142.5, 142.0, 135.3, 134.8, 128.3, 128.2, 127.9, 127.4, 125.7, 122.0, 118.0, 114.3, 109.5, 65.6, 55.2, 46.5, 35.5, 31.0, 27.4, 27.2.

### Synthesis of dibenzyl (1-phenethyl-2-(4-phenylbutyl)-1H-benzo[*d*]imidazol-5-yl) methyl phosphate 7

The compound **6** (0.15 g, 0.39 mmol) was reacted with dibenzyl *N*,*N*-diisopropylphosphoramidite (0.39 mL, 1.17 mmol) in the presence of ^1^ *H*-tetrazole (4.3 mL of 0.45 M in acetonitrile, 1.95 mmol) in dry DMF (5 mL) at 0°C. The reaction mixture warmed to room temperature and stirred for 3 h. After completion, reaction mixture was cooled to 0°C and slowly added ^t^BuOOH (0.4 mL of 5–6 M solution in *n*-decane, 2.34 mmol,), and stirring was continued for 1 h at 0°C. The residue was taken in ethyl acetate (50 mL), washed with saturated NaHCO_3_ and brine. The combined organic layers were dried over anhydrous Na_2_SO_4_ and concentrated in *vacuo*. The resulted crude residue was purified by using silica gel column chromatography (ethyl acetate/hexane = 2∶3) gave phosphoric ester **7** (0.20 g, 82% for two steps) as a liquid.

#### Spectral data

^1^H NMR (CD_3_OD, 300 MHz): δ 7.84 (d, 2H, *J* = 6.0 Hz), 7.78 (d, 1H, *J* = 6.3 Hz), 7.69 (d, 1H, *J* = 6.6 Hz), 7.60 (d, 1H, *J* = 6.3 Hz), 7.55 (d, 1H, *J* = 6.6 Hz), 7.27–7.21 (m, 4H), 7.19–7.10 (m, 8H), 6.98 (d, 1H, *J* = 6.0 Hz), 6.90 (d, 1H, *J* = 6.3 Hz), 5.60 (s, 2H), 5.11 (t, 4H, *J* = 5.4 Hz), 4.67 (t, 2H, *J* = 4.8 Hz), 3.26–3.16 (m, 4H), 2.77 (t, 4H, *J* = 6.0 Hz), 2.66–2.59 (m, 4H).

### Synthesis of (1-phenethyl-2-(4-phenylbutyl)-1H-benzo[*d*]imidazol-5-yl)methyl dihydrogen phosphate PLS5

The compound **7** (40 mg, 0.062 mmol) was treated with TFA (2 mL) in the presence of TIPS (0.5 mL) at 0°C. The reaction mixture was warmed to room temperature and stirred for 2 h. After completion, TFA was removed under vacuum and the resulted residue was dissolved in DCM (20 mL) and washed with saturated NaHCO_3_, H_2_O and brine. The combined organic layers were dried over anhydrous Na_2_SO_4_ and concentrated under *vacuo,* afforded crude product of PLS5 (26 mg, 90%). The crude residue was purified by using HPLC gave the pure product PLS5 as amorphous solid.

#### Spectral data

^1^H NMR (CD_3_OD, 300 MHz): *δ* 7.85 (s, 1H); 7.69 (d, 1H, *J* = 6.3 Hz), 7.54 (d, 1H, *J* = 6.3 Hz), 7.24–7.19 (m, 2H), 7.18–7.10 (m, 5H), 6.90 (d, 1H, *J* = 6.6 Hz); 5.60 (s, 2H), 5.11 (d, 2H, *J* = 5.4 Hz), 3.76 (s, 3H), 3.23 (t, 2H, *J* = 5.4 Hz), 2.61 (t, 2H, *J* = 5.7 Hz), 1.85–1.68 (m, 4H).; ^13^C NMR (CD_3_OD, 75 MHz): 161.5, 155.8, 142.8, 138.9, 133.1, 132.1, 129.6, 129.4, 127.1, 127.0, 126.5, 115.7, 113.9, 113.6, 67.7, 55.8, 36.1, 31.8, 27.3, 26.6.; ^31^P NMR (121 MHz, CD_3_OD): *δ* 0.48.

RP-HPLC R_t_ = 19.52 (linear gradient, 5–95% B, 30 min, 2.0 mL/min) and % of purity = 97.5; MS (MALDI-TOF) m/z = 481.27 [M+H] (calculated m/z = 480.18).

### Synthesis of (1-benzyl-2-(4-phenylbutyl)-1H-benzo[*d*]imidazol-5-yl)methyl dihydrogen phosphate PLS1

Compound PLS1 was synthesized using general procedure followed for the synthesis of PLS5.

#### Spectral data

^1^H NMR (CD_3_OD, 300 MHz): *δ* 7.82–7.60 (m, 2H), 7.26 (d, 1H, *J* = 6.0 Hz); 7.20–7.15 (m, 8H), 6.92 (dd, 2H, *J* = 3.0 and 6.0 Hz), 5.14 (d, 2H, *J* = 6.0 Hz), 4.67 (t, 2H, *J* = 6.0 Hz), 3.19 (t, 2H, *J* = 6.0 Hz), 2.66–2.58 (m, 4H), 1.63–1.53 (m, 4H). ^13^C NMR (CD_3_OD, 75 MHz): 155.6, 142.8, 138.8, 138.7, 138.3, 132.4, 131.8, 130.1, 129.5, 129.4, 128.6, 127.1, 126.6, 113.9, 113.5, 67.9, 36.1, 35.7, 31.9, 26.7, 26.0.;^ 31^P NMR (121 MHz, CD_3_OD): *δ* 0.43.

RP-HPLC R_t_ = 20.23 (linear gradient, 5–95% B, 30 min, 2.0 mL/min) and % of purity = 98.2, MS (MALDI-TOF) m/z = 451 [M+H] (calculated m/z = 450.17).

### Synthesis of (2-(4-cyclohexylbutyl)-1-phenethyl-1H-benzo[*d*]imidazol-5-yl)methyl dihydrogen phosphate PLS2

Compound PLS2 was synthesized using general procedure followed for the synthesis of PLS5.

#### Spectral data

^1^H NMR (CD_3_OD, 300 MHz); *δ* 7.85 (s, 1H), 7.82 (d, 1H, *J* = 3.0 Hz), 7.64 (d, 1H, *J* = 6.0 Hz). 7.24–7.12 (m, 3H), 6.96–6.94 (m, 2H), 5.16 (d, 1H, *J* = 6.0 Hz), 4.71 (t, 2H, *J* = 6.0 Hz), 3.23 (t, 2H, *J* = 6.0 Hz), 2.61 (t, 2H, *J* = 6.0 Hz), 1.72–1.51 (m, 5H), 1.32–1.27 (m, 2H), 1.23–1.14, (m, 8H), 0.93–0.87 (m, 2H). ^13^C NMR (CD_3_OD, 75 MHz); 155.9, 138.6, 138.5, 138.4, 132.5, 131.8, 130.1, 128.6, 126.7, 114.0, 113.5, 68.1, 68.0, 38.7, 37.9, 35.7, 34.5, 27.7, 27.5, 27.4, 26.1. ^31^P NMR (121 MHz, CD_3_OD); *δ* 0.21.

RP-HPLC R_t_ = 22.22 (linear gradient, 5–95% B, 30 min, 2.0 mL/min) and % of purity = 96.8; MS (MALDI-TOF) m/z = 471.33 [M+H] (calculated m/z = 470.23).

### Synthesis of (2-heptyl-1-(4-phenylbutyl)-1H-benzo[*d*]imidazol-5-yl)methyl dihydrogen phosphate PLS3

Compound PLS3 was synthesized using general procedure followed for the synthesis of PLS5.

#### Spectral data

^1^H NMR (CD_3_OD, 300 MHz); *δ* 7.81 (s, 1H), 7.23 (d, 1H, *J* = 6.3 Hz), 7.57 (d, 1H, *J* = 6.0 Hz), 7.21–7.20 (m, 2H), 7.14 (d, 3H, *J* = 6.0 Hz), 5.11 (d, 2H, *J* = 6.0 Hz), 4.42 (t, 2H, *J* = 6.0 Hz), 3.12 (t, 2H, *J* = 6.0 Hz), 2.67 (t, 2H, *J* = 6.0 Hz), 1.90–1.73 (m, 6H), 1.45–1.29 (m, 8H), 0.88 (t, 2H, *J* = 6.0 Hz). ^13^C NMR (CD_3_OD, 75 MHz); 154.0, 141.3, 137.4, 131.3, 130.5, 128.1, 128.0, 125.7, 125.0, 112.1, 66.3, 44.7, 34.6, 31.3, 28.8, 28.5, 28.2, 27.7, 26.4, 24.9, 22.2, 14.0. ^31^P NMR (121 MHz, CD_3_OD).*δ* 0.45.

RP-HPLC R_t_ = 19.82 (linear gradient, 5–95% B, 30 min, 2.0 mL/min), and % of purity = 97.2; MS (MALDI-TOF) m/z = 459.38 [M+H] (calculated m/z = 458.23).

### Synthesis of 1-(4-methoxybenzyl)-2-(4-phenylbutyl)-1H-benzo[*d*]imidazole-5-carboxylic acid 8

Compound **5** (0.2 g, 0.48 mmol) was treated with 4*N* HCl (5 mL) and refluxed for 2 h. After completion, the solvent was removed under *vacuo* and the resulted residue was dissolved in ethyl acetate (20 mL) and washed with saturated NaHCO_3_, H_2_O and brine. The combined organic layers were dried over anhydrous Na_2_SO_4_ and concentrated under *vacuo.* The crude residue was purified using silica gel column chromatography (MeOH/DCM = 1∶19) to afford 8 (0.15 g, 80%) as a white solid.

#### Spectral data

^1^H NMR (CDCl_3_, 300 MHz): δ 8.42 (s, 1H); 7.91 (d, 1H, *J* = 6.0 Hz); 7.24–7.22 (m, 3H); 7.15–7.11 (m, 3H); 6.84 (d, 1H, *J* = 9.0 Hz), 6.71 (d, 1H, *J* = 6.0 Hz), 5.21 (s, 2H), 3.73 (s, 3H), 2.68 (t, 2H, *J* = 6.0 Hz), 2.52 (t, 2H, *J* = 6.0 Hz), 1.90–1.84 (m, 2H); 1.76–1.68 (m, 2H); ^13^C NMR (CDCl_3_, 75 MHz): 166.6, 159.1, 156.8, 141.5, 142.2, 138.6, 128.1, 127.2, 127.1, 125.7, 124.1, 124.0, 120.7, 114.2, 109.0, 54.7, 51.8, 45.9, 35.6, 31.3, 26.9, 26.5.

### Synthesis of methyl 2-(4-methoxyphenyl)-1H-benzo[*d*]imidazole-5-carboxylate 12

Methyl 3,4-diaminobenzoate (0.61 g, 3.67 mmol) **10** was added to anisaldehyde **11** (0.45 mL, 3.67 mmol) in the presence of anhydrous *p*-TsOH (0.13 g, 0.73 mmol) in DMF (2 mL). The resulted reaction mixture was heated to 80°C and stirred for 1 h. The reaction mixture was cooled to 0°C and added saturated Na_2_CO_3_ solution dropwise with vigorous stirring. Then the mixture was treated with ethyl acetate (20 mL) and washed with H_2_O, brine and dried over anhydrous Na_2_SO_4_. The resulted organic layer was evaporated under vacuum to yield the crude product, which was purified using silica gel column chromatography (MeOH/DCM, 1∶49) afforded desired product **12** (0.83 g, 80%) as liquid.

#### Spectral data

^1^H NMR (CDCl_3_, 300 MHz); δ 8.31 (br s, 1H), 8.02 (d, 2H, *J* = 6.6 Hz), 7.97 (dd, 1H, *J* = 0.9 and 6.3 Hz), 7.62 (d, 1H, *J* = 6.3 Hz), 6.98 (d, 1H, *J* = 6.6 Hz), 3.94 (s, 3H), 3.86 (s, 3H). ^13^C NMR (CDCl_3_, 75 MHz): 167.7, 161.7, 154.0, 128.4, 124.6, 124.3, 121.6, 114.6, 55.4, 52.1.

### Synthesis of methyl 2-(4-methoxyphenyl)-1-(4-phenylbutyl)-1H-benzo[*d*]imidazole-5-carboxylate 13

The compound **12** (0.5 g, 1.77 mmol) was reacted with,4-phenylbutylbromide (0.31 mL, 1.77 mmol) in anhydrous DMF (10 mL) and K_2_CO_3_ (0.49 g, 3.54 mmol) was slowly added at 0°C. The mixture was warmed to 60°C and stirred for 2 h. After completion, reaction mixture was quenched with saturated NH_4_Cl solution and extracted with ethyl acetate (2×50 mL). The combined organic layers were washed with brine, dried over anhydrous Na_2_SO_4_, and concentrated in *vacuo*. The obtained residue was purified over silica gel column chromatography (ethyl acetate/hexane = 2∶3) gave non separable isomeric mixture **13** (0.67 g, 92%) as a colorless liquid.

#### Spectral data for isomers

^1^H NMR (CDCl_3_, 300 MHz): *δ* 8.49 (d, 1H, 0.6 Hz), 8.12 (d, 1H, 0.6 Hz), 8.01 (t, 1H, *J* = 1.5 Hz), 7.99 (t, 1H, *J* = 1.5 Hz), 7.79 (d, 1H, *J* = 6.3 Hz), 7.61 (dd, 4H, *J* = 4.0 and 6.3 Hz), 7.34 (d, 1H, *J* = 6.3 Hz), 7.28–7.23 (m, 5H), 7.20 (dd, 4H, *J* = 1.2 and 2.4 Hz), 7.18 (d, 1H, *J* = 2.4 Hz), 7.10–7.05 (m, 4H), 7.03 (d, 1H, *J* = 6.6 Hz), 4.30–4.20 (m, 4H), 3.97 (s, 3H), 3.95 (s, 3H), 3.89 (s, 6H), 2.57 (t, 1H, *J* = 5.4 Hz), 1.90–1.80 (m, 4H), 1.65–1.55. ^13^C NMR (CDCl_3_, 75 MHz); 167.7, 161.0, 156.3, 155.3, 146.6, 142.6, 141.3, 141.2, 138.8, 135.3, 130.7, 128.4, 128.3, 126.0, 124.2, 124.1, 122.3, 122.0, 114.3, 112.2, 109.3, 55.4, 52.1, 44.8, 35.0, 29.2, 29.0, 28.1.

### Synthesis of 2-(4-methoxyphenyl)-1-(4-phenylbutyl)-1H-benzo[*d*]imidazole-5-carboxylic acid 9

4*N* HCl (10 mL) was treated with compound **13** (0.6 g, 1.45 mmol and refluxed for 2 h. The reaction mixture was concentrated *under vacuum*, and the residue was dissolved in ethyl acetate (2×100 mL) and washed with saturated NaHCO_3_ and brine. The combined organic layers were dried over anhydrous Na_2_SO_4_ and concentrated under vacuum. The obtained crude residue was purified using silica gel column chromatography (MeOH/DCM, from 1∶20 to 1∶5) to yield the mixture of isomers **9a** and **9b** (0.5 g, 86%) as liquid.

#### Spectral data

^1^H NMR (CDCl_3_, 300 MHz); *δ* 7.86 (d, 1H, 6.0 Hz), 7.70 (t, 1H, 3.0 Hz), 7.60 (dd, 2H, *J* = 6.0 and 9.0 Hz), 7.22 (dd, 1H, *J* = 3.0 and 6.0 Hz), 7.16–7.14 (m, 2H), 7.12–7.10 (m, 3H), 7.03 (d, 1H, *J* = 6.0 Hz), 4.38–4.31 (m, 2H), 3.86 (s, 3H), 2.05 (t, 1H, *J* = 6.0 Hz), 1.71–1.66 (m, 2H), 1.48–1.45 (m, 2H). ^13^C NMR (CDCl_3_, 75 MHz); 169.1, 160.3, 154.6, 144.7, 142.1, 141.5, 137.9, 135.2, 1238.2, 128.1, 125.7, 123.6, 123.4, 122.5, 120.3, 117.8, 114.2, 112.0, 109.8, 55.3, 44.0, 34.2, 28.7, 27.7.

### Synthesis of methyl 1-(4-phenylbutyl)-1H-benzo[*d*]imidazole-5-carboxylate 16

Compound **15** (0.5 g, 2.84 mmol) was reacted with 4-phenylbutylbromide (0.5 mL, 2.84 mmol) in anhydrous DMF (10 mL) and K_2_CO_3_ (0.79 g, 5.68 mmol) was added slowly at 0°C. The resulting reaction mixture was warmed to 60°C and stirred for 2 h. After completion, reaction mixture was quenched with saturated NH_4_Cl, and extracted with ethyl acetate (250 mL). The combined organic layers were washed with brine, dried over anhydrous Na_2_SO_4_, and concentrated in *vacuo*. The crude residue was purified over silica gel column chromatography (MeOH/DCM = 1∶49) to yield the mixture of isomers **16** (0.79 g, 90%) as a colorless liquid.

#### Spectral data for isomers

^1^H NMR (CDCl_3_, 300 MHz); *δ* 8.53 (s, 1H), 8.10 (t, 2H, *J* = 6.0 Hz), 7.34–7.29 (m, 3H), 7.16–7.14 (m, 3H), 4.23–3.94 (m, 2H), 2.68–2.63 (m, 2H), 2.30–2.20 (m, 2H). ^13^C NMR (CDCl_3_, 75 MHz): 167.7, 147.3, 144.6, 139.9, 133.4, 128.7, 126.5, 124.8, 123.5, 122.8, 120.1, 112.1, 52.2, 44.5, 32.6, 30.9.

### Synthesis of 1-(4-phenylbutyl)-1H-benzo[*d*]imidazole-5-carboxylic acid 14

4*N* HCl (10 mL) was added to compound **16** (0.7 g, 2.27 mmol) in 1,4-dioxane (5 mL) and refluxed for 2 h. The reaction mixture was concentrated under vacuum and dissolved in ethyl acetate (20 mL). The mixture was washed with saturated NaHCO_3,_ brine and dried over anhydrous Na_2_SO_4._ The combined organic layer was concentrated under vacuum. The crude residue was purified using silica gel column chromatography (MeOH/DCM, from 1∶20 to 1∶5) to yield the mixture of isomers **14a** and**14b** (0.56 g, 84%) as liquid.

#### Spectral data for isomers

^1^H NMR (CDCl_3_, 300 MHz); *δ* 8.51 (s, 1H), 8.12 (t, 2H, *J* = 6.0 Hz), 7.32–7.21 (m, 3H), 7.17–7.13 (m, 3H), 4.21–3.93 (m, 2H), 2.65–2.61 (m, 2H), 2.32–2.23 (m, 2H). ^13^C NMR (CDCl_3_, 75 MHz): 166.7, 147.1, 144.2, 138.3, 134.6, 126.9, 125.6, 124.9, 123.5, 121.3, 111.1, 51.1, 45.8, 33.4, 29.6.

### Synthesis of *N*-phenethyl-5-phenylpentanamide 19

5-phenylpentanoic acid (1 g, 5.6 mmol) was reacted with 2-phenylethanamine (0.65 mL, 5.6 mmol) in the presence of coupling agents EDCI (1.12 g, 6.2 mmol), HOBt (0.84 g, 6.2 mmol) and Et_3_N (2.34 mL, 16.8 mmol) in dry DCM (30 mL) and stirred for 4 h. The reaction mixture was quenched with water and washed with saturated citric acid, NaHCO_3_ and brine. The combined organic layers were dried over anhydrous Na_2_SO_4_ and concentrated under vacuum. The crude residue was purified using silica gel column chromatography (ethyl acetate/hexane = 3∶7) to yield the amide **19** (1.42 g, 90% for two steps) as liquid.

#### Spectral data

^1^H NMR (CDCl_3_, 300 MHz); δ 7.32–7.21 (m, 5H), 7.21–7.13 (m, 5H), 5.37 (br s, 1H), 3.51 (q, 2H, *J* = 5.1 Hz), 2.80 (t, 2H, *J* = 5.4 Hz), 2.61 (t, 2H, *J* = 5.4 Hz); 2.13 (t, 2H, *J* = 5.1 Hz); 1.66–1.54 (m, 4H). ^13^C NMR (CDCl_3_, 75 MHz); 172.7, 142.2, 138.9, 128.7, 128.6, 128.4, 128.3, 126.5, 125.7, 40.4, 36.6, 35.7, 35.6, 31.0, 25.3.

### Synthesis of methyl 1-phenethyl-2-(4-phenylbutyl)-1H-benzo[*d*]imidazole-5-carboxylate 20

The compound **19** (0.55 g, 1.12 mmol) was reacted with methyl 4-chloro-3-nitrobenzoate (0.20 g, 0.93 mmol), in the presence of palladium trifluoroacetate (30 mg, 0.04 mmol), (*R*)-BINAP (56 mg, 0.04 mmol), and cesium carbonate (493 mg, 0.7 mmol) in dry toluene (10 mL). The reaction mixture was heated to 80°C under argon atmosphere and stirred for 12 h. After completion, reaction mixture was quenched with water (3 mL) and filtered through a pad of Celite. The filterate was washed with ethyl acetate (3×6 mL) and the collected organic layers was dried and evaporated. The resulted residue was dissolved in glacial acetic acid (20 mL) and was heated to reflux for 1 h in the presence of iron powder (0.63 g). The acetic acid was removed under vacuum, and the residue was suspended in saturated NaHCO_3_ and extracted with ethyl acetate (250 mL). The combined organic layers washed with brine and dried over anhydrous Na_2_SO_4_ and evaporated. The resulted crude residue was purified using silica gel column chromatography (ethyl acetate/hexane = 2∶3) to afford the desired product **20** (0.23 g, 60% for two steps) as a liquid.

#### Spectral data

^1^H NMR (CDCl_3_, 300 MHz); *δ* 8.37 (s, 1H), 7.90 (dd, 1H, *J* = 1.2 and 8.4 Hz), 7.24–7.14 (m, 6H), 7.12–7.05 (m, 3H), 6.89–6.83 (m, 2H), 4.24 (t, 2H, *J* = 6.9 Hz), 3.88 (s, 3H), 2.98 (t, 2H, *J* = 6.9 Hz), 2.56 (t, 2H, *J* = 7.5 Hz), 2.41 (t, 2H, *J* = 7.5 Hz), 1.77–1.67 (m, 2H), 1.64–1.54 (m, 2H).

### Synthesis of 1-phenethyl-2-(4-phenylbutyl)-1H-benzo[*d*]imidazole-5-carboxylic acid 17

4*N* HCl (10 mL) was added to compound **20** (0.7 g, 2.27 mmol) in 1,4-dioxane (5 mL) and refluxed for 2 h. The reaction mixture was concentrated under vacuum and dissolved in ethyl acetate (20 mL). The mixture was washed with saturated NaHCO_3_, brine and dried over anhydrous Na_2_SO_4._ The combined organic layer was concentrated under vacuum. The crude residue was purified using silica gel column chromatography (MeOH/DCM, from 1∶20 to 1∶5) to yield the desired product **17** (0.15 g, 80%) as a white solid.

#### Spectral data

^1^H NMR (CDCl_3_, 300 MHz); δ 8.69 (s, 1H), 8.10 (d, 1H, *J* = 5.4 Hz), 7.32–7.20 (m, 6H), 7.15 (d, 2H, *J* = 5.7 Hz), 6.94 (d, 2H, *J* = 4.5 Hz), 4.30 (t, 2H, *J* = 4.5 Hz), 3.04 (t, 2H, *J* = 4.5 Hz), 2.69–2.59 (m, 4H), 1.85–1.75 (m, 2H), 1.74–1.64 (m, 2H).; ^13^C NMR (CDCl_3_, 75 MHz): 171.0, 157.0, 142.1, 141.3, 137.6, 137.3, 128.9, 128.7, 128.4, 128.3, 127.2, 125.7, 124.5, 121.8, 109.0, 45.6, 35.9, 35.5, 31.0, 27.2, 26.7.

#### General procedures for the synthesis of bg-33, bg-1, bg-2, bg-5, bg-6, bg-27, bg-28, bg-29, bg-30 and bg-34 from acid derivatives

The molecules bg-33, bg-1, bg-2, bg-5, bg-6, bg-27, bg-28, bg-29, bg-30 and bg-34 were synthesized using the standard Fmoc-based, solid-phase peptide synthesis (SPPS) method using Rink amide resin, with an initial loading of 0.61 mmol/g, unless otherwise noted. Fmoc-Thr(PO(OBzl)OH)-OH and Fmoc-*β*-Ala-OH amino acids were purchased from Novabiochem. Resins were swollen in *N*,*N-*dimethylformamide (DMF) for 45 min prior to synthesis. For the bg-33, bg-1, bg-2, bg-27, bg-28, and bg-34 synthesis, only Thr(PO(OBzl)OH)-OH (5.0 eq.) was activated by treatment with HBTU (5.0 eq.) and HOBt (5.0 eq.) in DMF (2 mL) for 2 min. This solution was added to the free amine on resin, along with *N*,*N*-diisopropylethylamine (10.0 eq.), and the coupling reaction was allowed to proceed for 1 h with vortex stirring. After washing with DMF, Fmoc deprotection was achieved with 20% piperidine in DMF (1×10 min, 2×3 min). The resin was washed once again, and added synthesized acid derivatives in the presence of HBTU (5.0 eq.) and HOBt (5.0 eq.) along with *N*,*N*-diisopropylethylamine (10.0 eq.) continued vortex stirring 6 h. For the synthesis bg-5, bg-6, bg-29 and bg-30 initially coupled with Thr(PO(OBzl)OH)-OH followed by Fmoc-*β*-Ala-OH and then synthesized acid derivative were coupled. Finally, the resin was washed sequentially with DMF, methanol, dichloromethane, and ether, and then dried under a vacuum. Cleavage of the resin with TFA, TIPS and H_2_O afforded the molecules, which were purified by HPLC.

### Analytical HPLC conditions

The above synthesized molecules bg-33, bg-1, bg-2, bg5, bg-6, bg-2, bg-28, bg-29, bg-30 and bg-34 were purified to a minimum purity of 97% using a phenomenex column (C18, 250×10 mm, 5 micron), with a linear gradient from 5% aqueous acetonitrile (0.05% trifluoroacetic acid) to 95% acetonitrile (0.05% trifluoroacetic acid), over 35 min at a flow rate of 2.0 mL/min and detection at 230 nm (Table S2 in File S1).

### ELISA-based PBD-binding inhibition assay

A biotinylated p-T78 (DPPLHSpTAIYADEE-NH2) peptide was diluted with coating solution (KPL, Inc., Gaithersburg, MD) to the final concentration of 0.3 µM, and then 50 µL of the resulting solution was immobilized onto a 96-well, streptavidincoated plate (Nalgene Nunc, Rochester, NY). The wells were washed once with PBS + 0.05% Tween 20 (PBST), and incubated with 200 µL of PBS + 1% BSA (blocking buffer) for 1 h to prevent the unoccupied sites. Mitotic 293A lysates expressing HA-EGFP-Plk1 were prepared in the TBSN buffer (60 µg total lysates in 100 µL), applied onto the biotinylated peptide-coated ELISA wells immediately after mixing with the indicated amount of the competitor peptides, and then incubated with constant rocking for 1 h at 25°C. Next, the plates were washed four times with PBST, and to detect the binds HA-EGFP-Plk1, the plates were incubated for 2 h with 100 µL/well of anti-HA antibody at a concentration of 0.5 µg/mL in the blocking buffer, and then the plates were washed five times. Furthermore, the plates were incubated with 100 µL/well of secondary antibody at a 1∶1,000 dilution in the blocking buffer. Afterward, the plates were washed five times with PBST and incubated with 100 µL/well of 3,3′,5,5′-tetramethylbenzidine (TMB) substrate solution (Sigma, St. Louis, MO) until a desired absorbance was achieved. The reactions were terminated by the addition of 1 N H_2_SO_4_, and the optical densities were measured at 450 nm using an ELISA plate reader (Molecular Device, Sunnyvale, CA). Data are shown in Figures 3B and 6B.

### Molecular Modeling

All calculations were performed using Discover 2.98/InsightII with CVFF force field as described previously.^37^ The crystal structure of PBD of Plk1 having a pThr mimetic-containing C_6_H_5_(CH_2_)_8_− group binds was used as the computational model (3RQ7.pdb). The synthetic peptide inhibitor bg-34 was built from the coordinates of this pThr mimetic-containing C_6_H_5_(CH_2_)_8_− group as found in the crystal structure using the Builder module in Insight II. The computational complex model was solvated using a solvent sphere of water extending 30.0 Å around the phosphate atom of pThr. The system was initially minimized using 1000 steps of steepest decent and 3000 steps of conjugated gradient with a 14.0 Å nonbonded cutoff distance.

### Cellular uptake of bg-34

For evaluation of cellular uptake of bg-34, we tested with human cancer cell line, HeLa (human epithelial carcinoma cell line) that it was purchased from American Type Culture Collection (ATCC). HeLa cells were growth in Dulbecco's Modified Eagle Medium (DMEM, GIFCO BRL, Gaithersburg, MD, USA) that had been supplemented with 10% (v/v) fetal bovine serum (FBS) (Gibco, Gran Island, NY, USA), 100 U/mL of penicillin, and 100 µg/mL of streptomycin (Gibco) in 5% CO_2_ humidified air at 37°C. The uptake ability of Bg-34 on cancer cell lines was performed with FITC conjugated bg-34. 2×10^4^ cells/well of HeLa cells were prepared and cultured into 24-well polystyrene plates for 24 h. The cells were incubated for 3 h with 200 µM of FITC conjugated bg-34, then washed tree times with PBS, and mounted with mount solution (VECTOR Shield. VECTOR corp., USA). Cellular uptake of Bg-34 was imaged on a fluorescence microscope (OLYMPUS IX81, Olympus Inc, Japan). Images were processed using Image analysis program software (Metamorph, Molecular Devices Inc., USA).

### Morphological change by treatment of bg-34

2×10^4^ cells/well of HeLa cells were cultured into 24-well plates for 24 h and incubated for 24 h after treatment with 300 µM of bg-34. The morphological change of HeLa cells by Bg-34 was showed after fixation with 4% paraformaldehyde (PFA, Biosesang Inc., Seongnam, Republic of Korea). The morphological change was imaged on a microscope (OLYMPUS IX81, Olympus Inc, Japan), and its images were processed using image analysis program software (Metamorph, Molecular Devices Inc., USA).

### Apoptotic Effect

To confirm the apoptotic effect by treatment of bg-34, the compound was added in Hela cells with 300 µM concentration, and incubated for 24 h. For staining, cover-slip was fixed with 4% PFA and was stained with 100 µg/mL of Acridine orange/ethidium bromide solution (Sigma Aldrich, St. Louis, USA) for 15 min at 37°C. The cover-slips were washed tree times with PBS. The result of staining was imaged on a fluorescence microscope using a blue filter. Images were processed using Image analysis program.

### PLK-1 kinase Inhibition Test

The kinase inhibition effect of PLK-1 by treatment of bg-34 was studied using the CycLex Polo-like kinase Assay/Inhibitor Screening Kit (MBL Inc., Japan). 2.5×10^5^ cells/well of HeLa cells were cultured in 6-well in media with 300 µM of bg-34 for 12 h. The 30 µg/100 µL of total proteins were purified from cancer cells were mixed with kinase reaction buffer, was incubated at 30°C for 30 min. For detection of kinase activity of PLK-1, samples were incubated for 1 h with anti-phospho-serine/threonine polyclonal antibody PPT-07 solution, for 1 h with HRP-conjugated Anti-rabbit IgG, and for 10 min with substrate reagent solution. Finally, stop solution was added into each well. Measure absorbance of each well was read using a spectrophotometric plate reader at 450 nm/560 nm.

## Supporting Information

File S1 **Contains the files: Figure S1** Modeling structure of the Plk1-PBD, which shows the presence of the phosphate binding pocket, pyrrolidine binding pocket and Try rich hydrophobic channel. **Figure S2** Solid phase synthesis of FITC conjugated derivative of bg-34. **Table S1** Inhibitory activity of Plk1 PBD by compounds PLHSpT, PPG, PLS1-PLS3, PLS5, Bg-1, Bg-2, bg-27- Bg-30, Bg-33 and Bg-34. **Table S2** HPLC retention time, % of purity and MALDI-TOF mass values of bg-33, bg-1, bg-2, bg5, bg-6, bg-2, bg-28, bg-29, bg-30 and bg-34 molecules.
(DOC)
