# Supplementary material for: Design and Synthesis of a Cell-Permeable, Drug-Like Small Molecule Inhibitor Targeting the Polo-Box Domain of Polo-Like Kinase 1
Source: PLoS One. 2014 Sep 11;9(9):e107432. doi: 10.1371/journal.pone.0107432 (PMC4161390; doi:10.1371/journal.pone.0107432)
Supplement: File S1 — Contains the files: Figure S1 Modeling structure of the Plk1-PBD, which shows the presence of the phosphate binding pocket, pyrrolidine binding pocket and Try rich hydrophobic channel. Figure S2 Solid phase synthesis of FITC conjugated derivative of bg-34. Table S1 Inhibitory activity of Plk1 PBD by compounds PLHSpT, PPG, PLS1-PLS3, PLS5, Bg-1, Bg-2, bg-27- Bg-30, Bg-33 and Bg-34. Table S2 HPLC retention time, % of purity and MALDI-TOF mass values of bg-33, bg-1, bg-2, bg5, bg-6, bg-2, bg-28, bg-29, bg-30 and bg-34 molecules. (DOC) [file pone.0107432.s001.doc]

**SUPPLEMENTARY MATERIALS**

[**Design and Synthesis of a Cell-Permeable, Drug-Like Small Molecule Inhibitor Targeting the Polo-Box Domain of Polo-Like Kinase 1**](http://www.ncbi.nlm.nih.gov/pubmed/23498919)

Ganipisetti Srinivasrao^1¶^, Jung-Eun Park^2¶^, Sungmin Kim^1¶^, Mija Ahn^1^, Chaejoon Cheong^1^, Ky-Youb Nam^3^, Pethaiah Gunasekaran^4^, Eunha Hwang^1^, Nam-Hyung Kim^4^, Song Yub Shin^5^, Kyung S. Lee^2^, Eunkyung Ryu^1^*, Jeong Kyu Bang^1^*

*^1^Division of Magnetic Resonance, Korea Basic Science Institute, Ochang, Chung-Buk, Republic of Korea; ^2^Laboratory of Metabolism, Center for Cancer Research, National Cancer Institute, National Institutes of Health, Bethesda, MD, USA; ^3^Institute for Innovative Cancer Research and Department of Convergence Medicine, Asan Medical Center, Seoul, Republic of Korea; ^4^Molecular Embryology Laboratory, Department of Animal Sciences, Chungbuk National University, Cheongju, Chung-Buk, Republic of Korea; ^5^Department of Bio-Materials, Graduate School and Department of Cellular & Molecular Medicine, School of Medicine, Chosun University, Gwangju, Republic of Korea*

**Keywords**

Polo-like kinase 1, Polo-box domain (PBD), Cell permeable, Apoptosis, Inhibitor

*Corresponding authors:

Jeong Kyu Bang, Ph.D.

Korea Basic Science Institute

Ochang, Chungbuk 363-883, Cheongwon

Republic of Korea

Tel: +82-43-240-5023, Fax: +82-43-240-5059

E-mail: [bangjk@kbsi.re.kr](mailto:bangjk@kbsi.re.kr)

^¶^These authors contributed equally to this work.

**
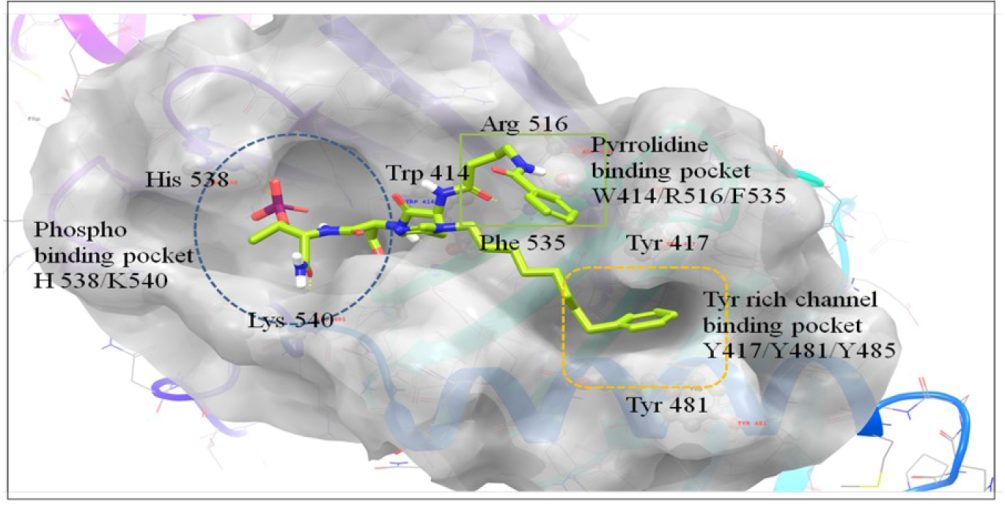
**

**Figure S1.** Modeling structure of the Plk1-PBD, which shows the presence of the phosphate binding pocket, pyrrolidine binding pocket and Try rich hydrophobic channel.


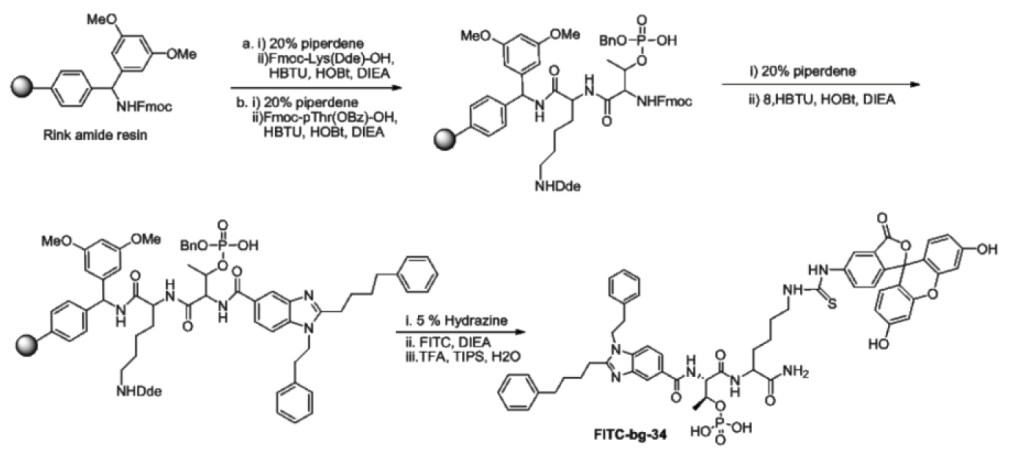


**Figure S2**. Solid phase synthesis of FITC conjugated derivative of bg-34

| **Molecules** | **PBD inhibitory activity^a^** | **IC50 (µM)** |
| --- | --- | --- |
| PLHSpT | high | 23.42 |
| Bg-34 | strong | 4.53 |
| PPG  PLS5  Bg-5  Bg-6 | moderate  weak  weak  weak |  |
| PLS1  PLS2  PLS3  Bg-33  Bg-1  Bg-2  Bg-27  Bg-28  Bg-29  Bg-30 | not determind  not determind  not determind  not derermind  not derermind  not derermind  not derermind  not derermind  not derermind  not derermind |  |

^a^ Measured by an ELISA-based Plk1 PBD competition assay.

**Table S1**: Inhibitory activity of Plk1 PBD by compounds PLHSpT, PPG, PLS1-PLS3, PLS5, Bg-1, Bg-2,bg-27- Bg-30, Bg-33 and Bg-34.

| Molecules | Acid derivative of benzimidazle used | Purity of the molecules | HPLC retention times(min) | Expected mass value | Observed mass value |
| --- | --- | --- | --- | --- | --- |
| Bg-33 | 7 | 97.9 | 19.50 | 594.22 | 595.21 |
| Bg-1 | 12 | 96.2 | 18.02 | 580.21 | 581.11 |
| Bg-2 | 12 | 98.0 | 18.47 | 580.21 | 581.22 |
| Bg-27 | 15 | 96.4 | 12.87 | 474.17 | 475.18 |
| Bg-28 | 15 | 97.8 | 13.27 | 474.17 | 475.23 |
| Bg-5 | 12 | 96.8 | 17.98 | 651.25 | 652.18 |
| Bg-6 | 12 | 96.0 | 18.42 | 651.25 | 652.33 |
| Bg-29 | 15 | 97.2 | 16.44 | 545.52 | 546.28 |
| Bg-30 | 15 | 98.5 | 13.80 | 545.52 | 546.28 |
| Bg-34 | 8 | 96.7 | 22.96 | 578.23 | 579.30 |
| Bg-34-FITC | 8 | 95.8 | 23.80 | 1096.15 | 1098.45 |

**Table S2:** HPLC retention time, % of purity and MALDI-TOF mass values of bg-33, bg-1, bg-2, bg5, bg-6, bg-2, bg-28, bg-29, bg-30 and bg-34 molecules.
